# Unraveling Belowground Community Assembly in Temperate Steppe Ecosystems

**DOI:** 10.3390/biology14101350

**Published:** 2025-10-02

**Authors:** Ping Wang, Shuai Shang, Zhengyang Rong, Jingkuan Sun, Jinzhao Ma, Zhaohua Lu, Fei Wang, Zhanyong Fu

**Affiliations:** 1 Shandong Key Laboratory of Eco-Environmental Science for the Yellow River Delta, Shandong University of Aeronautics, Binzhou 256603, China; wangpingcumtb@126.com (P.W.); shangshuai8983@126.com (S.S.); sunjingkuan@126.com (J.S.); mjz167448612@163.com (J.M.); luzhaohua@cumtb.edu.cn (Z.L.); 2 Institute of Restoration Ecology, China University of Mining and Technology-Beijing, Beijing 100083, China; rzy2579@gmail.com (Z.R.); lanyou401@163.com (F.W.); 3 State Key Laboratory of Water Resource Protection and Utilization in Coal Mining, Beijing 102209, China

**Keywords:** root traits, assembly pattern, spatial differentiation, driving factors, temperate steppe

## Abstract

**Simple Summary:**

This study presents a large-scale field survey of the temperate steppes of Inner Mongolia, introducing a novel framework to classify vegetation based on root traits. The concept of root importance value was introduced, incorporating the architectural and functional indices of belowground roots, as well as the root life form spectrum. The present research reveals that these steppes can be categorized into three distinct community subtypes (meadow, typical, and desert steppe) and thirteen plant associations. The analysis uncovered a clear spatial gradient in root strategies, defining three ecological adaptation types. Furthermore, the key environmental drivers for each community were identified. Precipitation and soil nutrients are identified as key drivers shaping the spatial distribution of belowground root traits in meadow steppe. Water limitation was central in typical steppe, while desert steppe was influenced by altitude and temperature. This work provides a significant advance in understanding steppe ecosystems from a belowground perspective.

**Abstract:**

The composition, architecture, and plant traits of temperate steppe communities are intricately associated with environmental factors. However, most studies primarily focus on aboveground observations, often overlooking the critical role of belowground root systems. Here we conducted a field survey at a large-regional scale to investigate the composition of temperate steppe communities and plant root traits. Cluster analysis, correspondence analysis and Pearson correlation coefficient matrix method were employed to classify vegetation associations based on plant community composition and root traits. The principal driving and limiting factors shaping plant root communities were systematically investigated. The results showed that the temperate steppe was categorized into three community subtypes: meadow steppe, typical steppe, and desert steppe, comprising five plant groups and thirteen plant associations. The RLFS analysis, based on belowground architectural and functional traits, demonstrated a spatial gradient differentiation with three ecological adaptations: tufted herbs, rhizome herbs, and non-tufted or rhizome herbs. Key environmental driving factors for meadow steppe included precipitation, soil carbon, nitrogen, and phosphorus content, while the average growing-season temperature as a limiting factor. The environmental driving factors for the typical steppe were not apparent, and the limiting factor was water. For the desert steppe, the environmental driving factors were altitude and average growing-season temperature. These findings reveal notable spatial heterogeneity and a distinct distribution pattern in community composition and vegetation classification based on belowground root traits in the Inner Mongolia steppes.

## 1. Introduction

Grassland ecosystems are the largest types of terrestrial ecosystems globally, holding substantial economic value through grass-based products and significant ecological functions, such as water conservation, biodiversity preservation, windbreaks, and sand fixation, and climate regulation [1]. The Inner Mongolian grassland, a principal component of the temperate grasslands spanning the Asian-European continent, serves as a crucial ecological barrier against soil erosion and desertification and a vital livestock production base in North China [2,3,4]. Additionally, its extensive distribution, significant east–west expanse, distinct hydrothermal gradient, varied soil types, and the presence of three grassland types (meadow, typical, and desert steppes) make it an ideal site for ecological research.

The assembly of plant community is a dynamic process that involves the filtering of species from a regional pool into specific local environments through multi-layer environmental conditions and biological interactions [5,6]. This process is influenced by a complex interplay of long-term environmental factors, including spatial heterogeneity, climate, soil characteristics, and disturbance regimes, as well as the intricate interactions among organisms within the communities [7]. As a result, the composition, architecture, and functional attributes of plant communities are shaped by these multifaceted drivers. The temperate steppe, a primary natural ecological landscape in Eurasia, exemplifies this complexity. Due to its environmental heterogeneity, the temperate steppe hosts a wide array of vegetation subtypes, forming complex steppe communities. These communities are not only diverse but also exhibit unique adaptations to their local environmental conditions, making them a valuable model system for studying the interplay between environmental factors and community structure. However, recent global climate change and the intensification of human activities have introduced new challenges to these ecosystems. These changes have substantially altered the composition, architecture, and dynamics of steppe communities [8]. Therefore, exploring plant community composition and architecture under varying environmental conditions, and the relationship between plant traits and ecosystems is crucial for predicting community response to climate change [9].

Over their long evolutionary history, plants have developed unique adaptive strategies. Previous research on the composition, architecture, and function of plant communities has predominantly focused on aboveground aspects, such as species diversity [10], productivity [11], functional groups [12,13,14] and stability in specific regions [15,16,17]. In contrast, belowground communities have remained relatively underexplored. Plant roots possess specific architectures-such as length, depth, width, volume, diameter, density, and biomass of roots-that enable them to adapt to varying spatial conditions and resource availability. Root traits play a critical role in shaping the species composition and architecture of plant communities [18,19]. However, existing studies on plant roots have primarily concentrated on underground biomass [20], root turnover [19,21,22], soil elements [23,24,25], and root traits under controlled conditions [26,27].

Recent research has been conducted on the belowground root systems of natural grasslands both domestically and internationally. These studies have yielded reliable data, significantly enriching our understanding of root ecology and underground ecology [20,28]. Root functional traits play a crucial role in regulating the functions of ecosystems [29]. In particular, in steppe ecosystems, these underground traits can serve as vital indicators of ecosystem resilience and maintenance capacity under environmental changes. To further explore these dynamics, we conducted a large-regional scale field survey across a temperate steppe region. Our objectives were to investigate the composition and architecture of plant communities and their root traits, elucidate the spatial differentiation patterns of belowground root systems, identify the influencing factors of spatial differentiation, and reveal the spatial distribution patterns of root traits under diverse environmental conditions. This study aims to address two key questions: (1) What are the patterns of plant community assembly based on belowground root characteristics? (2) Are there cooperative adaptations between plant roots and the environment?

## 2. Materials and Methods

### 2.1. Study Area

**Figure 1:**
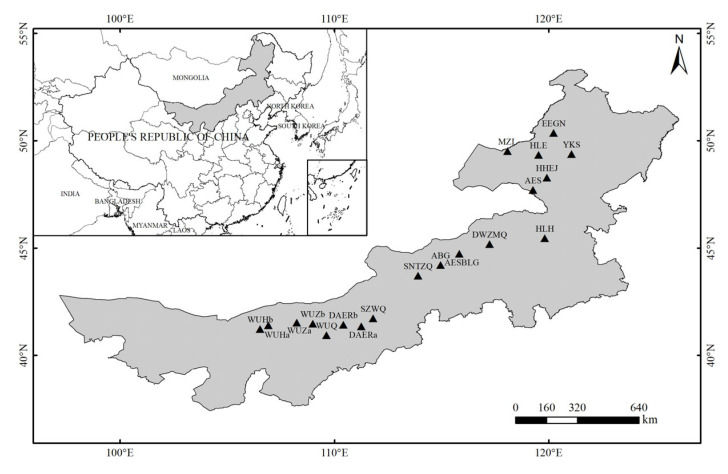
The geographic locations of study areas.

The study area is located in the temperate steppe of Inner Mongolia, China (40°55′–50°21′ N, 106°26′–121°4′ E), with elevations ranging from 556 m to 1741 m (Figure 1). The region experiences a temperate monsoon climate characterized by short, hot summers with concentrated precipitation and long, cold winter with snowfall in some areas. The average annual temperature varies between −2.7 °C and 8.4 °C, and the average annual precipitation ranges from 134 mm to 441 mm across the region.

Meteorological data from the China Meteorological Data Service Center (CMDSC, http://data.cma.cn/site/index.html (accessed on 31 December 2019)), which contain the average annual precipitation (YR), the average annual temperature (YT), the accumulated precipitation during the growing season (June–August, HMR), the average monthly temperature during the growing season (June–August, HMT), the average monthly temperature during the cold months (December–February, CMT).

The soil in the eastern part of the area is primarily black calcic, and the western part mainly consists of brown calcic loam and brown calcic sandy soil. These climatic and soil conditions jointly contribute to the various subtypes of temperate steppe, including meadow steppe with dominant species of *Stipa baicalensis*, *Leymus chinensis* and *Carex pediformis*, and typical steppe with *Stipa krylovii*, *L. chinensis*, *Allium polyrhizum*, etc. Desert steppes are built upon species like *Stipa glareosa* and *Stipa breviflora*.

Given the terrain and community vegetation types in this region, sampling sites were selected according to the administrative division through Hulun Buir, Xilingol League, Ulanqab City, Baotou City, Bayannur, and other places from northeast to southwest, with a distance of about 50–100 km between adjacent sampling sites. From northeast to southwest Inner Mongolia, the study sites were Ergun City (EEGN), Manzhouli City (MZL), Hailar District (HLE), Yakeshi City (YKS), Honghua Erji (HHEJ), Arxan City (AES), Huolin Gol City (HLH), East Ujimqin Banner (DWZMQ), Aershanbaolige (AESBLG), and Abaga Banner (ABG), Sonid Left Banner (SNTZQ), Siziwang Banner (SZWQ), Darhan-Muminggan Joint County (DAERa, DAERb), Urat Front Banner (WUQ), Urat Middle Banner (WUZa, WUZb), and Urat Back Banner (WUHa, WUHb), a total of 19 study sites were set up. The field vegetation survey and sampling work were carried out during the growing season of steppe plants in July and August of 2018 and 2019. Each sampling site had three representative sampling plots with flat terrain and uniform vegetation communities and three parallel quadrats were sampled within every plot, totaling 57 sampling plots and 171 quadrats. The sampling transect spans approximately 10 degrees of latitude and 15 degrees of longitude, extending from northeast to southwest.

### 2.2. Field Surveys and Index Determination

**Figure 2:**
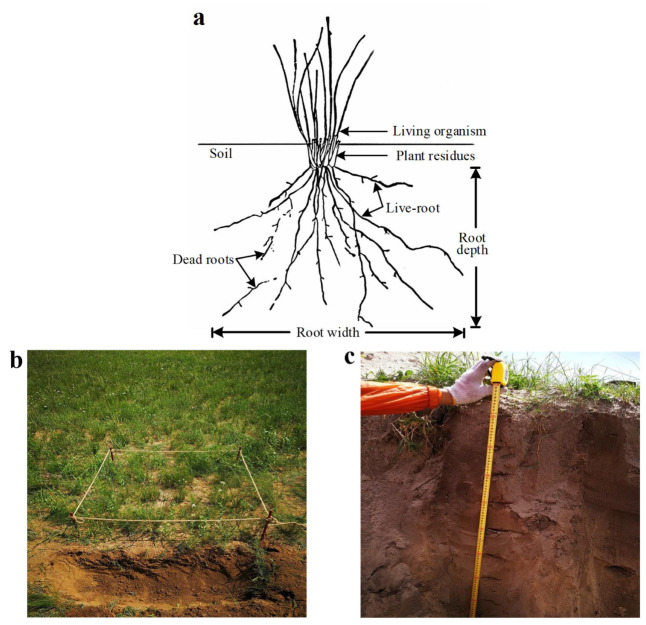
Description of root characteristics measurement (**a**), root acquisition (**b**) and digging depth (**c**).

Field surveys were conducted from late July to early August in 2018 and 2019. The longitude (long), latitude (lati), and altitude (alti) of each sampling plot were recorded. Within each plot, three 1 m × 1 m quadrats were established for community surveys. Within each quadrat, a comprehensive inventory was taken, meticulously recording the species, height, coverage, and abundance of the vegetation present. Subsequently, a soil profile, exceeding 1 metre, was carefully excavated along the periphery of one side of the quadrat using a spade, and then all plant specimens within the quadrat were systematically excavated, commencing from the periphery and progressing towards the centre, and endeavoring to minimize damage to the primary root systems. In accordance with the excavation depth, an in-depth quantification was conducted on the root length, root width, and root depth of five representative plants in their natural habitat. The root length was meticulously delineated as the maximal length of the root segments, with the root depth and width visually represented in Figure 2. Other root samples were promptly packed into sizable, resealable plastic bags for preservation, and subsequently conveyed to a provisional research station for meticulous sorting by species. Initially, these samples were submerged in plastic containers, facilitating the comprehensive immersion required to dislodge soil, detritus, and other particulate matter adhering to the plant roots. Next, the samples were thoroughly rinsed with copious amounts of water to ensure root cleanliness, and any necrotic roots were excised. The roots of all plants in the quadrats were excavated, cleaned, and classified. The average root length (RL), root width (RW), and root volume (RV) were measured and calculated. Additionally, the above- and below-ground parts of the plants were separated. The fresh weight was gauged. Then, the samples were placed into an envelope, and transported to the laboratory, where they were dried at 65 °C to a constant weight (±0.05 g). Subsequently, the belowground biomass (BGB) of each species was identified. The architectural and functional indices of the roots were constructed and calculated as detailed in the following [30]:

(1) $$\mathrm{Total}\;\mathrm{root}\;\mathrm{length}/(m\cdot m^{-2}):\;TRL=\sum_{i=1}^{S}RLi\times Ni$$

(2) $$\mathrm{Total}\;\mathrm{root}\;\mathrm{width}/(m\cdot m^{-2}):\;TRW=\sum_{i=1}^{S}RWi\times Ni$$

(3) $$\mathrm{Total}\;\mathrm{root}\;\mathrm{volume}/(m^{3}\cdot m^{-2}):\;TRV=\sum_{i=1}^{S}{\left(RWi/2\right)}^{2}\times \pi \times RDi\times Ni$$

(4) $$\mathrm{Total}\;\mathrm{belowground}\;\mathrm{biomass}/(g\cdot m^{-2}):\;TB=\sum_{i=1}^{S}BGBi$$

where *S* represents the species richness in the quadrat, where species *i* is located, and *Ni* represents the abundance of *i*. *RLi*, *RWi* and *RDi* represent the average root length, root width and root depth of *i*, respectively, and *BGBi* represents the belowground biomass of species *i*.

To identify the dominant species within the community, the index known as the Root Important Value (*RIV*) was established to assess the degree of the dominance in the underground community. The *RIV* for species *i* in a quadrat was defined as follows:

(5) $$\mathrm{Architecture}\;\mathrm{Index}:\;CI=\frac{RLi}{TRL}+\frac{RVi}{TRV}$$

(6) $$\mathrm{Function}\;\mathrm{Index}:\;FI=\frac{BGBi}{TB}$$

(7) Number Index: NI=Pi

(8) $$\mathrm{The}\;\mathrm{RIV}\;\mathrm{of}\;\mathrm{the}\;\mathrm{species}\;i:\;RIVi=\sqrt{CI^{2}+FI^{2}+NI^{2}}$$

**Figure 3:**
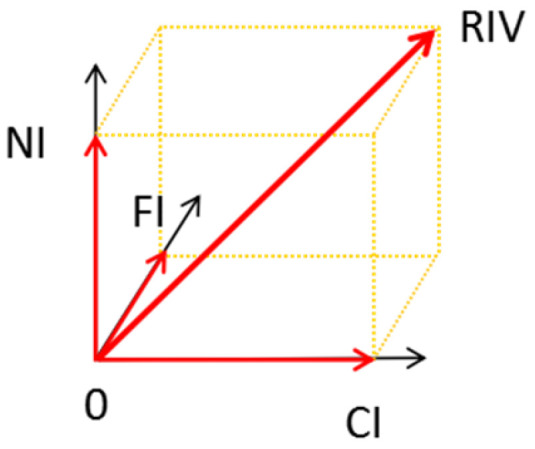
Schematic diagram of the Root Important Value.

It represents the vector sum of the architectural, functional and quantitative traits of species in the subterranean space (Figure 3). *RIV* for all species in the quadrat was calculated and sorted to determine the dominant species within the community for each plot.

Meanwhile, several diversity indices and formulas as follows [30]:

(9) Species Richness: Sr=S

(10) $$\mathrm{Margalef}\;\mathrm{Index}:\;MI=\frac{\left(S-1\right)}{Ln(N)}$$

(11) $$\mathrm{Simpson}\;\mathrm{Index}:\;D=1-\sum_{i=1}^{S}Pi^{2}$$

(12) $$\mathrm{Shannon}-\mathrm{Wiener}\;\mathrm{Index}:\;H=-\sum_{i=1}^{S}Pi\times Ln(Pi)$$

(13) $$\mathrm{Pielou}\;\mathrm{Index}:\;E=\frac{H}{Ln(S)}$$

(14) $$N\;\mathrm{represents}\;\mathrm{the}\;\mathrm{total}\;\mathrm{abundance}\;\mathrm{of}\;\mathrm{the}\;\mathrm{plants}\;\mathrm{in}\;\mathrm{quadrat}:\;N=\sum_{i=1}^{S}Ni$$

(15) $$\mathrm{Pi}\;\mathrm{is}\;\mathrm{on}\;\mathrm{behalf}\;\mathrm{of}\;\mathrm{the}\;\mathrm{relative}\;\mathrm{abundance}:\;Pi=\frac{Ni}{N}$$

Additionally, the mixed sampling method was employed both within and around each quadrat, obtaining soil samples at 0–30 cm depth, with 10 cm increments per layer in sections. The mixed soil samples were taken back to the laboratory for drying. Then, they were ground through a 60-mesh sieve. Total carbon (TC) and total nitrogen (TN) in the soil were determined using an elemental analyzer, and total phosphorus (TP) was assessed by the molybdenum antimony resistance colorimetric method.

### 2.3. Statistical Analysis

Cluster analysis of community composition, architecture, and root traits was performed. Some sample objects were set as initial centers, and the distance between each remaining object and the nearest center was calculated iteratively. This process continued until the sum of squared errors reached a local minimum, indicating optimal clustering. During the iteration, the positions of the clustering centers were constantly updated to minimize the overall distance. This allows for the identification of common characteristics among different communities. The Pearson correlation coefficient matrix was used to clarify strong correlations between community indices and environmental factors. Consequently, the effects of individual environmental factors on community composition, root architectural and functional traits, associated processes, mechanisms, and relative contributions were investigated. Correspondence analysis was adopted to explore the relationship between environmental variables across sampling sites. Variables with similar characteristics had smaller category distances, facilitating the analysis of the relationship between environmental variables. The common underground characteristics of plant communities in analogous environments were identified, as well as the primary and secondary factors driving underground community assembly across large-scale regions.

Statistical data analysis was conducted using R version 3.5.2, clustering analysis was performed using the ggplot2 and the factoextra packages, the Pearson correlation coefficient matrix analysis was carried out using the ggcorrplot package, and correspondence analysis was implemented using the ca package. All statistical tests were fulfilled at a significance level of α = 0.05. Figures were generated using the plot, the ggplot 2 package, and Origin 2019.

## 3. Results

### 3.1. Community Cluster

**Figure 4:**
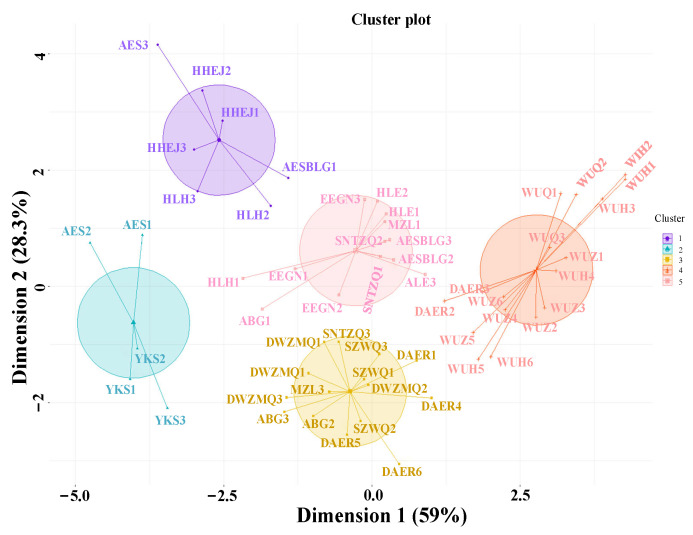
Cluster analysis of plant communities of 19 sampling sites.

Based on the community diversity index and root traits (Table 1), the clustering results were classified. As shown in Figure 4, the 19 sampling sites in this study are divided into the following five groups, with their specific characteristics described as follows:

(1) Meadow-hybrid group (AES, YKS):

The meadow-hybrid group exhibited the highest average value of the community root architectural index, abundant belowground biomass (TB_a_ = 400.76 ± 150.1), the highest species richness per quadrat (Sr_a_ = 19 ± 1), and high community evenness (D_a_ = 0.715 ± 0.109). The dominant species in the community were *L. chinensis* and *C. pediformis*. The secondary dominant species were likely *S. baicalensis* and *Elymus dahuricus*.

(2) Meadow steppe group (HLH, HHEJ):

The meadow steppe group was characterized by rich belowground biomass (TB_a_ = 435.42 ± 112.96), a high species richness index (Sr_a_ = 15.5 ± 1.5), and low evenness due to the dominance of one or two species (D_a_ = 0.576 ± 0.124). The dominant species in the community included *C. pediformis*, *L. chinensis* and possibly *S. baicalensis*. The secondary dominant species was *Cleistogenes caespitosa*, among others.

(3) Meadow-typical group (EEGN, HLE, AESBLG, SNTZQ):

This group encompassed both meadow steppe and typical steppe community types, characterized by similar root architecture, function indices (TB_a_ = 186.79 ± 31.4), and diversity indices such as the species richness index (Sr_a_ = 12 ± 2). In the meadow community, the dominant species were *C. pediformis*, and *Carex duriuscula*, with *S. baicalensis* as the secondary species. In the typical steppe community, *S. krylovii* was the dominant species, though it was replaced by *A. polyrhizum* and *Cleistogenes squarrosa* in some plots.

(4) Typical steppe group (ABG, DWZMQ, SZWQ, MZL, DAERa, DAERb):

In this group (Sr_a_ = 12 ± 3), the dominant species was *S. krylovii*, and the secondary was *L. chinensis*. Additionally, *C. squarrosa*, *Convolvulus ammannii*, or *A. polyrhizum* appeared as secondary species or companion species in some sampling plots. The community exhibited high similarities in architectural traits.

(5) Desert steppe group (WUQ, WUZa, WUZb, WUHa, WUHb):

This group exhibited closely related community characteristics (Sr_a_ = 5 ± 2), as well as root architectural and functional traits (TB_a_ = 38.67 ± 21.9, TRV_a_ = 0.21 ± 0.17). The dominant species included *S. glareosa* or *S. breviflora*, and some sampling plots were dominated by *Allium mongolicum* and *C. ammannii*.

### 3.2. Vegetation Association Classification

Following the principle of similar combination, a total of 57 sampling plots’ communities were grouped into 13 vegetation associations based on actual conditions, such as species composition and dominant or sub-dominant species within each plot. These associations are as follows:

(1) Ass. *Stipa glareosa*-*Allium mongolicum*

It included three plots, characterized by the common dominant species of *S. glareosa* and *A. mongolicum* and the secondary species of *Asparagus dauricus*. The soil types in these plots were calcisols and arenosols with low organic matter content. This association presented low species richness (Sr_a_ = 3.5 ± 0.5) and biomass.

(2) Ass. *Stipa glareosa*-*Eragrostis ferruginea*

It comprised three plots with *S. glareosa* and *E. ferruginea* as the dominant species and *Artemisia scoparia* as the secondary species. The soil type was calcisols and arenosols with low organic matter content. This association exhibited medium species richness (Sr_a_ = 7.5 ± 0.5) but low evenness (D_a_ = 0.142).

(3) Ass. *Stipa krylovii*-*Carex duriuscula*-*Cleistogenes squarrosa*

It encompassed eight plots with the common dominant species of *S. krylovii*, *C. duriuscula*, *and C. squarrosa*, and the secondary species of *L. chinensis*. The primary soil types were kastanozems and chernozems. This association showed abundant organic matter, high species richness (Sr_a_ = 11 ± 1), and medium evenness (D_a_ = 0.545 ± 0.035).

(4) Ass. *Stipa krylovii*-*Salsola collina*-*Neopallasia pectinata*

It included three plots dominated by *S. krylovii*, *S. collina*, and *N. pectinata*, with *L. chinensis* as a secondary species. The soil type was predominantly calcisols with low organic matter content. These plots had medium species richness (Sr_a_ = 9.5 ± 0.5) and high evenness (D_a_ = 0.723).

(5) Ass. *Stipa breviflora*-*Convolvulus ammannii*

It comprised six plots, characterized by the dominant species of *S. breviflora* and *C. ammannii*, and the secondary species of *A. mongolicum*. The soil type was mainly calcisols with low organic matter content. Some plots exhibited gravelly surfaces. The richness and evenness of species were low (Sra = 4.5 ± 0.5, Da = 0.464 ± 0.075) as well as biomass.

(6) Ass. *Stipa krylovii*-*Salsola collina*-*Cleistogenes squarrosa*

It included three plots with the common dominant species of *S. krylovii*, *S. collina*, and *C. squarrosa*, and the secondary species of *L. chinensis* and *C. ammannii*. The soil type was primarily calcisols with low organic matter content. These plots exhibited high species richness (Sr_a_ = 11) but low evenness (D_a_ = 0.467).

(7) Ass. *Stipa breviflora*-*Eragrostis ferruginea*

It consisted of three plots dominated by *S. breviflora* and *E. ferruginea*, with the secondary species of *A. scoparia*. The soil types were primarily calcisols and arenosols. This association presented low species richness (Sr_a_ = 6.5 ± 0.5) and medium evenness (D_a_ = 0.544).

(8) Ass. *Leymus chinensis*-*Carex duriuscula*

It included a single plot with the dominant species of *L. chinensis* and *C. duriuscula*, and the secondary species of *S. baicalensis* and *A. sacrorum*. The soil type was mainly chernozems with high organic matter content. This plot exhibited high species richness (Sr_a_ = 14) and evennes (D_a_ = 0.741), and significant biomass.

(9) Ass. *Allium polyrhizum*-*Stipa krylovii*

It included two plots, characterized by the dominant species of *A. polyrhizum* and *S. krylovii* and the secondary species of *L. chinensis* and *A. ramosum*. The soil type was primarily kastanozems with medium soil organic matter. These plots showed high species evenness (D_a_ = 0.635).

(10) Ass. *Stipa krylovii*-*Leymus chinensis*

It included eleven plots dominated by *S. krylovii* and *L. chinensis.* Depending on the degree of degradation, the secondary dominant species varied, including *C. ammannii*, *C. squarrosa*, *A. ramosum*, *A. bidentatum*, *C. duriuscula*, *N. pectinata*, and others. The soil type was mainly kastanozems. The plots showed high species richness (Sr_a_ = 13 ± 2) and evenness (D_a_ = 0.7 ± 0.1).

(11) Ass. *Carex pediformis*-*Stipa baicalensis*

It consisted of nine plots with *C. pediformis* and *S. baicalensis* as the dominant species. The secondary dominant species, such as *Agropyron cristatum*, were present but less prominent. The soils type was mostly chernozems with high organic matter. These plots displayed high species richness (Sr_a_ = 16 ± 2) but low evenness (D_a_ = 0.5) due to the high level relative abundance of the dominant species.

(12) Ass. *Leymus chinensis*-*Elymus dahuricus*

It included three plots dominated by *L. chinensis* and *E. dahuricus*. The secondary dominant species varied, mainly including *P. bifurca.* The soil type was mainly chernozems with high organic matter. This association exhibited the highest species richness and evenness (Sr_a_ = 20, D_a_ = 0.823).

(13) Ass. *Stipa baicalensis*-*Leymus chinensis*-*Cleistogenes caespitosa*

It comprised two plots with the dominant species of *S. baicalensis*, *L. chinensis* and *C. caespitosa* and the secondary dominant species of *Potentilla.* The soil type was chernozems with high organic matter. These plots had high species richness and evenness (Sr_a_ = 13.5 ± 0.5, D_a_ = 0.68 ± 0.05).

### 3.3. Life Form Spectrum Analysis

**Figure 5:**
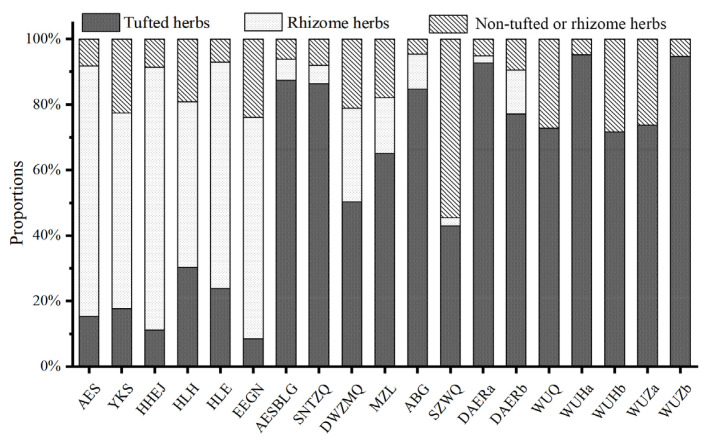
The root spectrum of community in Inner Mongolia steppe.

Based on the root adaptation characteristics of dominant species (*Stipa*, *Leymus*, *Carex*, etc.), we put root functional trait (such as the belowground biomass) as the indicator, the proportions of these three life forms in the study area were as follows (Figure 5).

(1) Tufted herbs: Plants that grow in small and dense clumps or tufts, typically with discoid roots (such as *Stipa* and *Allium*). This group included eight sampling sites, where tufted herbs dominated (R_a_ = 0.733), such as *S. krylovii* and *A. polyrhizum*. The proportion of rhizome herbs was dramatically reduced. Most of these sampling sites were located in the central part of the study area, within the typical steppe.

(2) Rhizome herbs: Plants with rhizomes that grow horizontally, connecting below ground while appearing separate above ground (such as *Leymus* and *Carex*). The area consisted of six sampling sites, with communities dominated by rhizome herbs (R_a_ = 0.673), such as *L. chinensis* and *C. pediformis*. Rhizome herbs accounted for more than 50%, while the proportions of non-tufted or rhizome herbs to tufted herbs was roughly equal. These sites were located in the eastern part of the temperate steppe and belonged to the meadow steppe vegetation subtypes.

(3) Non-tufted or rhizome herbs: Other herbs that do not exhibit tufted or rhizomatous growth forms. This type consisted of five sampling sites in the desert steppe of the western region, where tufted herbs also dominated (R_a_ = 0.816), including *S. glareosa* and *S. breviflora*, but rhizome herbs were rarely present in this group.

In summary, the proportions of species’ life forms and their geographic locations exhibited a distinct spatial differentiation pattern. However, the communities in the MZL were more similar to typical steppes, and notably differed from those in the eastern regions. Additionally, the high proportion of non-tufted or rhizome herbs in the SZWQ region was attributed to the degradation of typical communities.

### 3.4. Single-Factor Analysis and Correspondence Analysis of Influencing Factors

**Figure 6:**
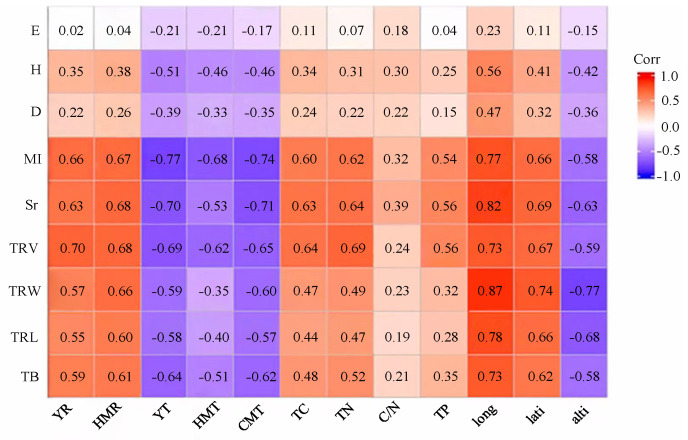
Pearson correlation coefficients between functional indicators and environmental factors. E: Pielou index; H: Shannon-Wiener index; D: Simpson index; MI: Margalef index; Sr: species richness; TRV: total root volume; TRW: total root width; TRL: total root length; TB: total belowground biomass; YR: the average annual precipitation; HMR: the accumulated precipitation during the growing season (June–August); YT: the average annual temperature; HMT: the average monthly temperature during the growing season (June–August); CMT: the average monthly temperature during the cold months (December–February; TC: total carbon; TN: total nitrogen; C/N: carbon-to-nitrogen ratio; TP: total phosphorus; long: longitude; lati: latitude; alti: altitude.

This study considered nine indices related to community composition and architecture, as well as root architectural and functional traits, alongside twelve environmental variables (including water, heat, soil and space). Pearson correlation analysis was conducted on the resulting 9 × 12 coefficient matrix, identifying strongly correlated indices for further single-factor analysis (Figure 6).

**Figure 7:**
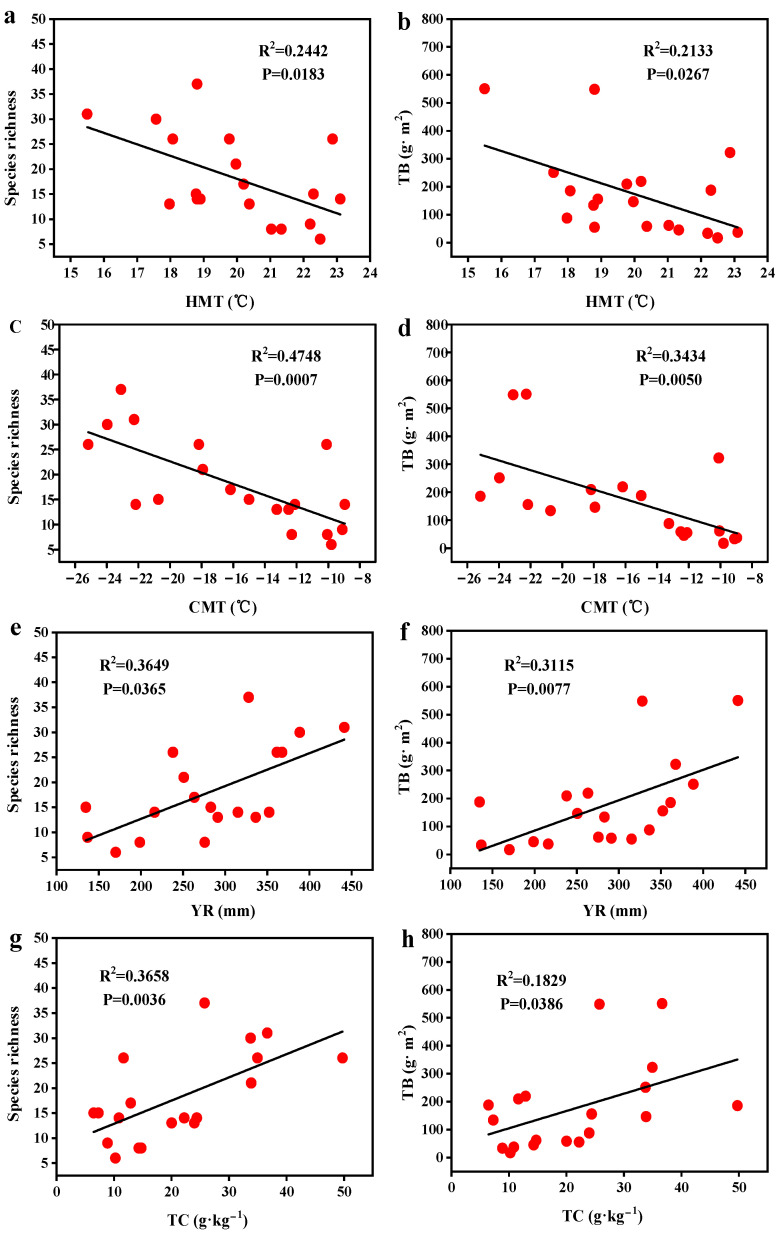
Correlations between environmental factors and species richness and belowground biomass. (**a**,**b**) represent the correlation analyses of HMT with species richness and TB, respectively; (**c**,**d**) represent the correlation analyses of CMT with species richness and TB, respectively; (**e**,**f**) represent the correlation analyses of YR with species richness and TB, respectively; (**g**,**h**) represent the correlation analyses of TC with species richness and TB, respectively. HMT: the average monthly temperature during the growing season (June–August); CMT: the average monthly temperature during the cold months (December–February); YR: the average annual precipitation; TC: total carbon; TB: total belowground biomass.

According to Pearson correlation analysis, Sr and TB were ultimately selected as community-dependent variables, with HMT, CMT, YR, and TC as explanatory factors of ecological environment variables for single-factor analysis (Figure 7).

The results revealed that both species richness and belowground biomass decreased with the increase in the average temperature during the growing season (June–August), and they increased as the average temperature dropped during the cold months (December–February) (*p* < 0.05). This indicated that excessively high temperatures in summer and insufficiently low temperatures in winter are detrimental to the growth and development of individual plants and the formation of community diversity. Conversely, species richness and belowground biomass elevated with annual precipitation and total soil carbon content (*p* < 0.05), indicating that these factors positively influence the growth and development of individuals plants and community diversity.

**Figure 8:**
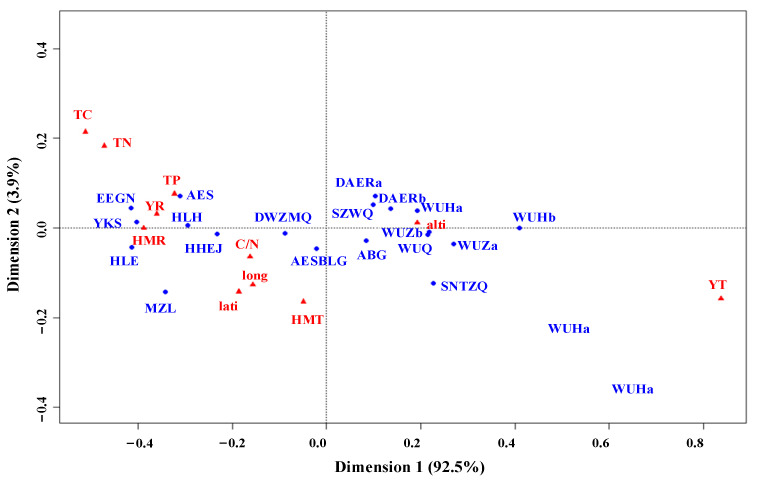
Correspondence analysis of environmental factors in Inner Mongolia steppe. The red triangles represented the environmental variables, and the blue dots were on behalf of the study sites.TC: total carbon; TN: total nitrogen; TP: total phosphorus; C/N: carbon-to-nitrogen ratio; HMR: the accumulated precipitation during the growing season (June–August); HMT: the average monthly temperature during the growing season (June–August); YT: the average annual temperature; lati: latitude; long: longitude; alti: altitude.

The analysis involved eleven environmental variables, such as spatial, climatic, and soil factors. The results are analyzed as follows (Figure 8). The first dimension explains most variables, accounting for 92.5%. Among them, adjacent sites exhibit high similarity in environmental conditions. Environmental variables with coincident directions have similar effects on a site. The closer a particular environmental variable is to a site, the stronger its impact on the site. From left to right, the arrangement generally follows a gradient of longitude and latitude from east to west across the study area. The blue dots on the left correspond to the meadow steppe in the eastern region, while those on the right are associated with the desert steppe in the western. The central region represents the typical steppe vegetation subtype or a transitional type.

Precipitation and soil phosphorus content are the primary environmental drivers in the meadow steppe, whereas the typical steppe shows no clear dominant factors. Altitude is the primary driving factor in the desert steppe. Longitude, latitude, average temperature during the growing season, and the soil carbon-to-nitrogen ratio are secondary environmental factors across almost all sampling sites. Concurrently, in the eastern region, soil total carbon and total nitrogen content, as secondary factors, have a significantly greater impact on the meadow steppe than on the typical steppe and desert steppe. The average annual temperature plays a minor role in explaining the composition, architecture, and function of vegetation communities in the study area.

## 4. Discussion

### 4.1. Root Traits and Spatial Distribution Patterns at the Community Scale

The spatial differentiation of belowground root systems in plant communities is a critical manifestation of ecological adaptation, primarily reflected in architectural and functional traits that serve as proxies for resource acquisition strategies. Root-level traits provide profound insights into how plant communities respond to and shape their environments. At a large spatial scale, Zhao investigated 532 plant quadrats of temperate steppe and found that most functional groups of temperate steppe in northern China were mainly composed of perennial forbs, rhizomatous grass, and bunchgrass at the functional group level, showing an obvious precipitation gradient effect [31]. What is more, our earlier work also proved that plant communities exhibit a distinct spatial gradient, transitioning from species-rich meadow steppe to typical steppe and desert steppe in the temperate steppe of Inner Mongolia [30]. The present study strengthens this framework by revealing how community assembly mechanisms and environmental filtering drive root trait distributions. In the meadow steppe, high productivity and stochastic assembly maintain elevated species richness (Sr_a_ = 17 ± 3). The dominance of rhizome herbs contributes to consistently high root architectural and functional indices, suggesting trait convergence under abundant resources, possibly due to facilitative interactions or niche complementarity. The typical steppe, with intermediate species richness (Sr_a_ = 12 ± 3), is dominated by tufted grasses, such as *Stipa* and *Cleistogenes*. In some sampling plots, they were replaced by *A. polyrhizum* and *C. squarrosa*. This variability in community composition corresponds to divergent root traits, reflecting differential responses to the degree of degradation. In the desert steppe, harsh conditions result in low productivity and species richness (Sr_a_ = 5 ± 2), accompanied by diminished root trait indices. Tufted grasses, such as *S. glareosa* and *S. breviflora*, also dominate here, while rhizome herbs are absent-a pattern indicative of strong abiotic filtering for conservative, drought-resistant root strategies.

In summary, a clear trend emerges: rhizome herbs dominate the humid northeast, while tufted herbs prevail in arid central and western regions. This shift underscores that tufted species possess traits aligned with drought tolerance, such as deeper rooting and higher root width [32]. Such trait-environment matching illustrates how functional adaptations facilitate coexistence and enhance ecosystem resilience. Ultimately, the synergistic interaction between species traits and habitats emerges as a central mechanism promoting adaptation across environmental gradients. This finding have also been confirmed by other scholars, confirming that root spatial pattern is a ubiquitous yet context-dependent feature of vegetation response to climate variability [33,34]. Future studies should integrate quantitative root architecture modeling with long-term soil moisture and nutrient monitoring to better resolve the causality of observed trait distributions. Furthermore, could provide deeper mechanistic insights into community–scale root adaptation.

### 4.2. Driving Factors of Spatial Distribution Patterns

Environmental factors impact community species composition, architecture, and root traits and functions in indirect or latent ways, as well as shape plant composition and community distribution. Local climate change and anthropogenic disturbances can lead to environmental variations [35], potentially driving divergence in community traits. Moreover, the interpretation of community characteristics based on sampling data can be occasionally one-sided and imperfect and distorted in some locations. Therefore, a corresponding analysis was conducted to explore the influence of similar natural environments on community assembly. In this study, the absence of meadow steppe at the MZL site, which is now dominated by typical steppe, may be attributed to shifts in local climate, soil, and other environmental conditions linked to urbanization [36].

The assembly of herbaceous communities based on traits is a deterministic process shaped by environmental filtering and similarity limitations [37]. Their effects vary across different scales. At broader regional scales, environmental filtering plays a dominant role, whereas similarity limitation is more critical at local scales [38]. In our study, three vegetation subtypes exhibit high similarity within each subtype, resulting in convergent community architectural and functional traits at a large-regional scale. In contrast, variations in composition and specific community traits at local scales, such as local dominant species, abundance, and proportion between plots, may be attributed to the similarity limitation caused by interspecific competition [35].

In the eastern region of the study area, ample precipitation and soft soil with high organic matter content create a wide ecological niche. These conditions facilitate the spread of rhizome herbs and the development of meadow steppe communities with abundant biomass [39]. The apoptosis of plants at low temperatures in winter enhances soil organic matter content, preserves propagules, and maintains the diversity and productivity within these communities [40]. Therefore, precipitation and soil nutrients are key environmental drivers in the eastern meadow steppe, while the average temperature during the growing season constrains community growth and development. In the central region of the study area, the average temperature rises during the growing season, leading to a gradual decrease and eventual disappearance of rhizome herbs. Tufted herbs with discoid roots demonstrate greater adaptability to the prevailing conditions, and moisture becomes the primary limiting factor in this regional environment [28]. By comparison, altitude exerts an environmental filtering effect on community assembly in the western region [41]. Low precipitation levels, sandy soil with minimal organic matter content, and elevated soil surface temperature during the growing season constitute challenging conditions for water retention. Consequently, these factors result in consistently low species richness, sparse community coverage, and significantly reduced root trait indices. Both precipitation and the average temperature during the growing season were identified as critical limiting factors.

The convergence and divergence of plant traits within communities are highly scale-dependent, and are related to the level and intensity of competition [42,43]. Trait convergence in similar environments is usually driven by the exclusion of species with less competitive traits. For instance, the formation of *Stipa*-dominated communities in typical steppe gives rise to various associations [44]. On the other hand, belowground biomass, as one of the few functional indicators of the underground part of plant community, has different responses to different environmental factors. Huang et al. studied the belowground roots of temperate grasslands in Inner Mongolia and found that total root biomass was positively correlated with annual precipitation and soil total nitrogen, and negatively correlated with average annual temperature [45]. In this study, it was found that the belowground biomass of plant communities was positively correlated with precipitation and negatively correlated with average annual temperature, which was basically consistent with the conclusions of previous studies [45,46]. It is essential to account for spatial and temporal variations to fully understand community assembly and succession processes [47]. Although current studies on root trait distributions provide insights into community assembly and diversity maintenance, they lack strong predictive capabilities. Future research should not only clarify the patterns of plant trait distribution within communities but also delve into the underlying coexistence mechanisms shaped by these traits.

## 5. Conclusions

This study employes a novel framework that integrates root traits with community composition and architecture to elucidate the mechanisms governing the assembly of plant root communities. The findings demonstrate that classifying vegetation based on belowground root traits offers a more accurate representation of spatial distribution patterns in steppe ecosystems. Analysis of species life form spectrum, grounded in the belowground architectural and functional traits of plant communities, highlights distinct ecological adaptations among three root system types in the study area: tufted herbs, rhizome herbs, and non-tufted or rhizome herbs. These systems exhibit clear spatial gradient differentiation. Furthermore, precipitation and soil nutrients are identified as key drivers shaping the spatial distribution of belowground root traits across different steppe types, whereas mean annual temperature, bulk density, and pH serve as major limiting factors. These insights enhance our understanding of the spatial differentiation of belowground root systems in plant communities within Inner Mongolia steppe. Meanwhile, this study also establishes a theoretical foundation for biodiversity conservation and vegetation restoration in these ecologically significant regions. Subsequent research should integrate aboveground plant traits with root functional data to advance a more comprehensive understanding of above-below ground interactions in plant ecological strategies and community assembly.

## Figures and Tables

**Table 1:** Community diversity index and root traits of 19 sampling sites.

Index	Abbreviation	Average	Maximum	Minimum
Species richness	Sr	10.9	21	3
Margalef index	MI	1.474	2.969	0.400
Simpson index	D	0.556	0.863	0.040
Shannon-Wiener index	H	1.212	2.207	1.112
Pielou index	E	0.519	0.844	0.099
Total belowground biomass/(g·m^−2^)	TB	173.83	717.20	15.80
Total root length/(m·m^−2^)	TRL	177.18	544.91	12.20
Total root width/(m·m^−2^)	TRW	129.33	441.29	7.59
Total root volume/(m^3^·m^−2^)	TRV	3.13	19.22	0.03

## Data Availability

The data presented in this study are available on request from the corresponding author.
